# Ocular Toxoplasmosis Mimicking Lymphoma: Exploring the Correlation and Distinction

**DOI:** 10.7759/cureus.13014

**Published:** 2021-01-30

**Authors:** Omid Yazdanpanah, Lea M Monday, Sarvani Surapaneni, Vijendra Singh, Jie Chi

**Affiliations:** 1 Internal Medicine, Wayne State University School of Medicine, Detroit, USA; 2 Internal Medicine, Wayne State University Detroit Medical Center, Detroit, USA; 3 Internal Medicine, John D. Dingell VA Medical Center, Detroit, USA; 4 Hematology and Oncology, Karmanos Cancer Institute, Detroit, USA

**Keywords:** b-cell chronic lymphocytic leukemia (b-cll), ocular lymphoma, primary intraocular lymphoma, primary central nervous system lymphoma, ocular toxoplasmosis, toxoplasma gondii, posterior uveitis

## Abstract

We present a case of a 74-year-old woman with chronic lymphocytic leukemia (CLL) who presented with unilateral blurry vision that had progressively worsened over a few weeks. Ophthalmic examination revealed unilateral anterior chamber, vitreous body inflammation along with retinal infiltration which was initially diagnosed with posterior uveitis. Analysis of vitreous fluid aspiration was negative for bacteria, fungal and viral etiologies. Despite the broad-spectrum intraocular antibiotics, her vision continued to decline, and she later developed retinal detachment. Cytology for lymphoma was negative. However, polymerase chain reaction (PCR) with internal transcribed spacer-specific (ITS) primer set detected *Toxoplasma gondii*, and the patient was diagnosed with intraocular toxoplasmosis. Treatment with systemic clindamycin, pyrimethamine, leucovorin, prednisone, and topical clindamycin for four weeks successfully prevented further ocular damage.

## Introduction

Toxoplasmic retinochoroiditis or ocular toxoplasmosis is an infective eye disease caused by a parasite, *Toxoplasma gondii*. It typically presents as recurrent posterior uveitis with altered vision and floaters. Patients with acquired immunodeficiency syndrome (AIDS) or individuals who are immunocompromised secondary to cancer and chemotherapy are at increased risk of this opportunistic infection [1]. Also, in patients with a history of lymphoma, ocular lymphoma is among differential diagnosis. Similar slit-lamp examination findings including retinal whitening and vitreous opacity make it even more challenging to distinguish between the malignant and infective processes [2]. Vitreous aspiration or retinal biopsy is usually required for culture, cytology, polymerase chain reaction (PCR), and flow-cytometry to define the final diagnosis [3,4]. Definitive diagnosis is demanding and is often made by delay. Although effective treatment options are available for both pathologies, a prolonged delay before diagnosis can lead to severe progression of the disease.

## Case presentation

**Figure 1:**
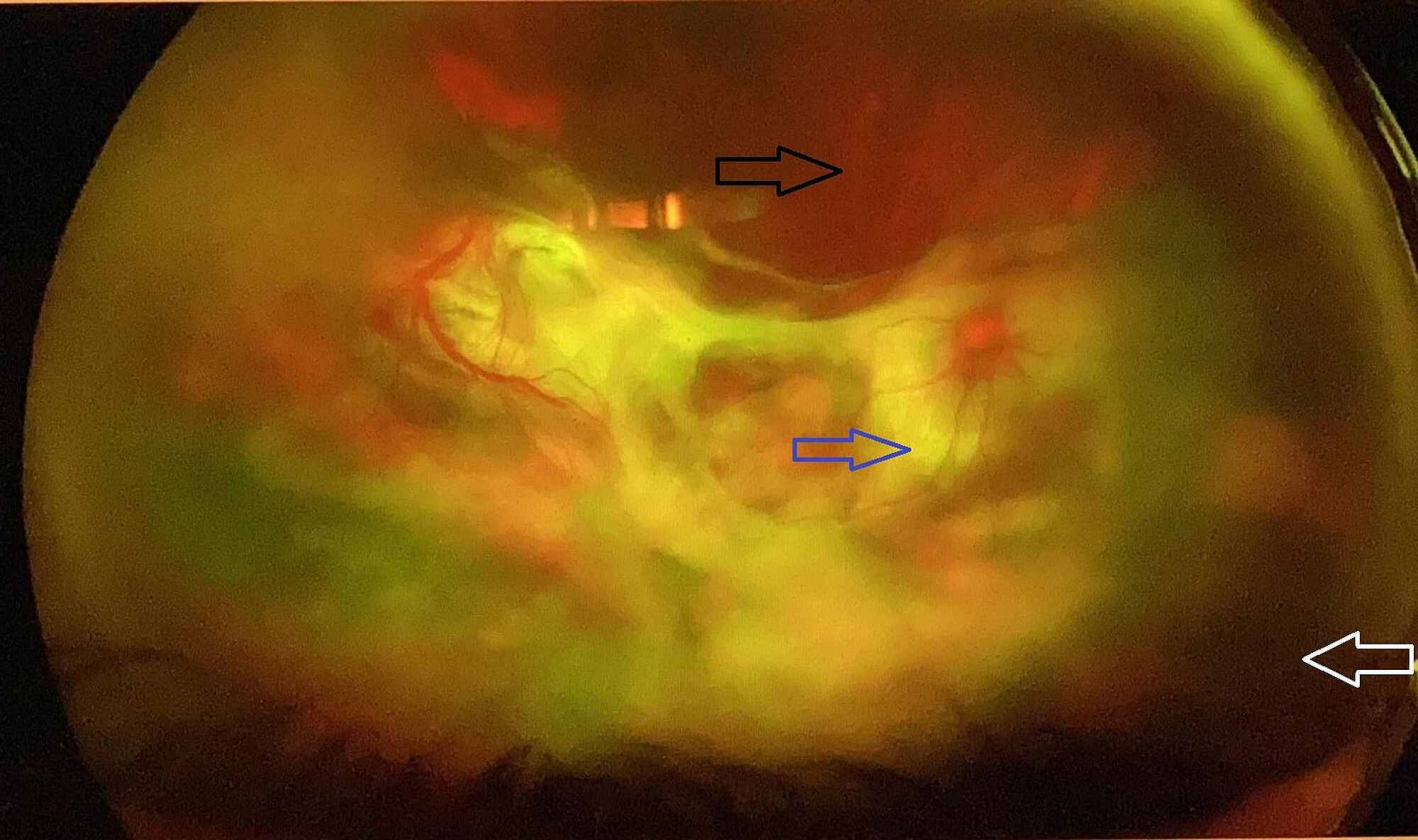
Right eye slit-lamp examination Retinal white lesions (blue arrow) are relatively masked by vitreous haziness due to inflammation and hemorrhage (black arrow). Peripheral retinal detachment (white arrow) is also observed.

The patient was a 74-year-old woman with chronic lymphocytic leukemia (CLL) who was initially treated with fludarabine, rituximab but had a relapse of disease and was treated with bendamustine and rituximab, ibrutinib, venetoclax. She had left mandibular lymph node biopsy confirmed transformation to non-germinal center diffuse large B-cell lymphoma (Richter’s transformation). Nearly 16 years after diagnosis, she was treated with Rituximab, Cyclophosphamide, Doxorubicin, Vincristine, and Prednisone (R-CHOP) for residual diffuse large B-cell lymphoma. One year later, was started back on ibrutinib 280 mg daily (lower dose because of side effect profile in past). Within two months of beginning the treatment, the patient started having symptoms of blurry vision in her right eye. It was not associated with any pain, redness, or photosensitivity. However, she had experienced floaters in the same eye before. Ophthalmologic examination showed severe visual impairment in the right eye that she could only detect hand motion. Intraocular pressure was 14 mmHg (normal range: 8 to 21 mmHg). Further examination revealed signs of inflammation in the anterior chamber and vitreous body with no hyphema. There was also a retinal white infiltration posteriorly. The left eye did not show any significant abnormality. These findings were consistent with the diagnosis of posterior uveitis (Figure 1).

**Figure 2:**
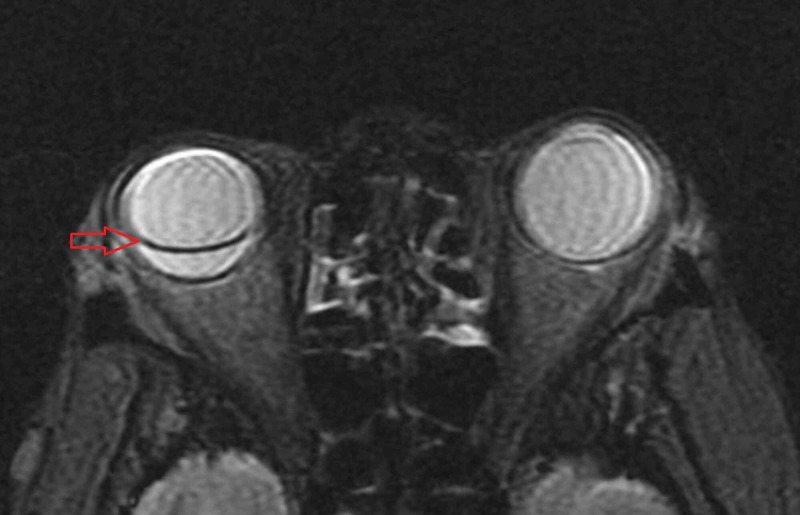
MRI-orbit Right eye retinal detachment (red arrow)

She underwent vitreous fluid aspiration and immediately was started on intraocular broad-spectrum antimicrobial treatment with ceftazidime, vancomycin, voriconazole, foscarnet, plus dexamethasone. The vitreous fluid was negative for lymphoma. Bacterial and fungal cultures, herpes simplex virus (HSV), varicella-zoster virus (VZV), and cytomegalovirus (CMV) PCR, serum galactomannan, and universal fungal PCR were all negative. However, the patient continued to deteriorate and began to experience eye pain. On follow-up eye examination, right retinal mass with a progressive retinal detachment was found. The left eye also showed small intraretinal lesion superior to optic nerve without overlying vitritis. Brain MRI with contrast confirmed right retinal detachment (Figure 2). Mild edema of the intraorbital and intraocular right optic nerve was also present. But, there was no intracranial mass lesion in favor of lymphoma on brain imaging.

**Figure 3:**
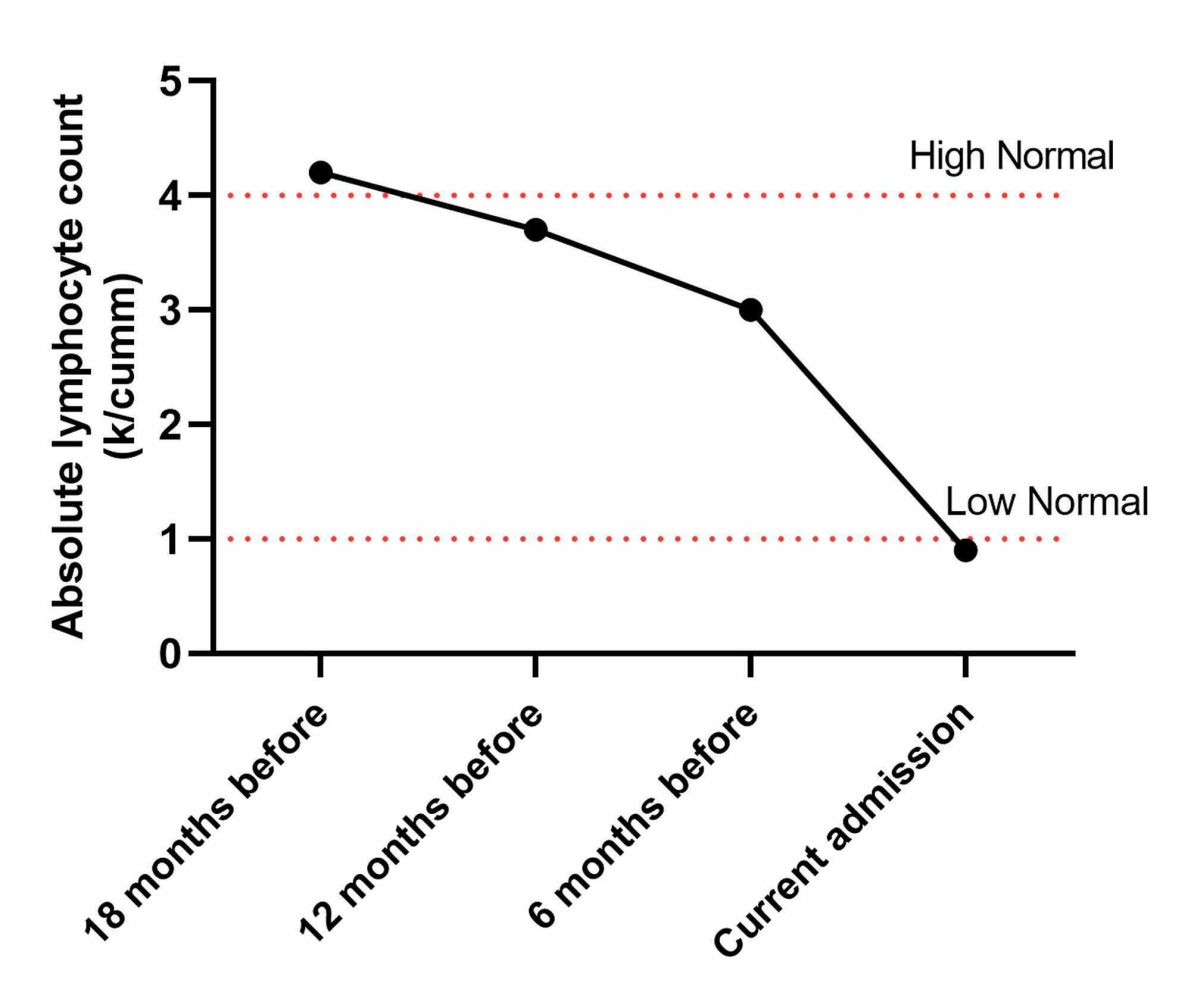
Patient’s average lymphocyte count over an 18-month time duration

A chorioretinal biopsy was performed because of clinical suspicion for vitreous lymphoma. Vitreous fluid flow cytometry showed a predominant T-cell population without aberrancy and insufficient B-cells. On the other hand, cytology was negative for abnormal lymphocytes which ruled out lymphoma. Nevertheless, PCR with internal transcribed spacer-specific (ITS) primer set detected *Toxoplasma gondii* DNA with greater than 5 million copies. Immunoglobulin G (IgG) for toxoplasmosis was also turned out positive. The patient’s lymphocyte count over an 18-month time duration is shown in Figure 3.

It confirmed the diagnosis of intraocular toxoplasmosis and the patient was started on treatment. As she was allergic to trimethoprim-sulfamethoxazole, she was started on systemic clindamycin and pyrimethamine accompanied by leucovorin. Given the severity of infection in the right eye lead to a rapid decline in vision, intraocular clindamycin was also started for both eyes. It was also accompanied by oral prednisone 60 mg daily to help to decrease optic nerve inflammation and to improve the vision in the right eye. After four weeks of treatment, she had no further deterioration of vision. She was started on atovaquone for prophylaxis.

## Discussion

Toxoplasmic retinochoroiditis or ocular toxoplasmosis is an inflammatory eye disease caused by *Toxoplasma gondii*. The majority of patients with ocular toxoplasmosis are asymptomatic. However, even those individuals will also have atrophic, well‐defined retinal scar without any history of active retinochoroiditis. That said, patients with active ocular disease usually present with posterior uveitis symptoms including floaters and altered vision. The vision loss can be permanent if it involves the macula. Nidus of fluffy white or yellow, necrotizing retinitis, or retinochoroiditis adjacent to a variably pigmented chorioretinal scar is the classic findings on examination [1]. Immunocompetent patients will usually have the active retinochoroiditis resolved, with or without treatment, within one to two months. Nonetheless, immunocompromised patients frequently show a relatively fulminant course [5]. Specifically, patients with AIDS can develop extensive areas of full-thickness retinal necrosis, panophthalmitis, and orbital cellulitis due to toxoplasmosis in spite of treatment [6].

CLL patients are at increased risk of opportunistic infections both due to disease biology and treatment. In patients with AIDS, the risk of toxoplasmosis reactivation increases with decreasing cluster of differentiation (CD)-4 cell count. Our patient was not lymphopenic for a prolonged duration, but it is likely that she has a lower T-cell count. Ibrutinib that is commonly used for the treatment of CLL in both newly diagnosed and relapsed cases, is also associated with an increased risk of opportunistic infections. Trimethoprim-sulfamethoxazole that is commonly used for *Pneumocystis jiroveci* pneumonia (PJP) prophylaxis, also provides protection from toxoplasmosis reactivation. Though PCP prophylaxis is not recommended for CLL patients who are on ibrutinib, still some centers use it. We do not routinely use PCP prophylaxis at our center for CLL patients who are on Ibrutinib [7,8].

In a patient with a history of lymphoma, primary or secondary ocular lymphoma is among differential diagnoses in a patient who presents with altered vision. Primary intraocular lymphoma (PIOL) is a subcategory of primary central nervous system lymphoma (PCNSL) in which lymphoma cells involves the eyes, with concomitant or subsequent intracranial involvement. Secondary intraocular lymphoma is a result of metastasis from the lymphoma originating outside the central nervous system (CNS). PCNSL is a rare malignancy but the incidence in the United States in the last 15 years has been tripled. It also can manifest with posterior uveitis symptoms [9]. The exact etiology of PIOL/PCNSL is not defined yet. Several hypotheses are proposed for this conundrum which some of them include a role for microorganisms. One speculation is that mutated Epstein-Barr virus (EBV) in immunocompromised patients, draws lymphocyte cells to the CNS and start the neoplastic transformation. EBV has been found in AIDS patients with PCNSL but is not cited in patients with PIOL [10]. The other organism that has been hypothesized to be involved in ocular lymphoma is *Toxoplasma gondii*. Shen et al. analyzed 10 cases of PIOL using microdissection and PCR. They found *Toxoplasma gondii* DNA in lymphoma cells but not in normal cells of two cases [11]. It has been suggested that infectious genes might incorporate in the B lymphocytes DNA and trigger the B-cell transformation and proliferation into particular B-cell clonal development and ultimately PIOL [12].

Ocular toxoplasmosis and lymphoma have shared slit-lamp examination findings, retinal whitening, and vitreous opacity. Therefore, distinguishing between these two to determine the underlying cause of posterior uveitis is challenging [2]. Vitreous aspiration or retinal biopsy is usually required in a combination with modalities including culture, cytology, and flow-cytometry to define the final diagnosis Exposure to steroids prior to biopsy can diminish the sensitivity of biopsy [3,13]. Nevertheless, the finding of genomic mutations such as MYD88 in the vitreous fluid has been detected in several intraocular lymphoma cases and can increase the sensitivity and specificity of biopsies [14]. The pathologic classification of PCNSL is the same as systemic non-Hodgkin lymphoma (NHL) and the most common subtype is diffuse large B cell lymphoma [15]. For ocular toxoplasmosis, serum and ocular fluid anti-Toxoplasma titers of IgM and IgG might be needed to support the diagnosis [4]. However, false-negative results have been reported specifically in CLL patients with hypogammaglobulinemia, due to the inability to produce IgG antibodies [16]. PCR of aqueous and vitreous samples for *Toxoplasma gondii* DNA has higher sensitivity and specificity to confirm the diagnosis, especially in immunocompromised patients. *Toxoplasma gondii* DNA was amplified in 75% of immunocompromised with ocular toxoplasmosis although sensitivity is lower, around 30%-40%, in immunocompetent cases. Despite low sensitivity, the specificity of PCR for toxoplasmosis is 100% [4,14].

Ocular toxoplasmosis in an immunocompetent patient will mostly self resolves within one to two months. Thus, risks vs benefits analysis for therapy is typically done on a case-by-case basis. However, being immunocompromised is generally considered as an indication for treatment. The goal of treatment is to limit the parasite multiplication during active retinitis as no antimicrobial medication has shown a cure for this infection. It is performed by classic therapy which includes pyrimethamine, sulfadiazine, or trimethoprim-sulfamethoxazole, and the addition of systemic corticosteroid [17]. Corticosteroid is being used for patients with significant vitreous inflammation and retinal vasculitis to help to preserve vision and enhance visual recovery. However, no definitive data has supported its efficacy [18]. On the other hand, treatment of PIOL is a combination of radiation, systemic, and intravitreal chemotherapy [9]. Ocular irradiation with prophylactic CNS treatment helps to control PIOL and prevent its spread to CNS [19]. High dose methotrexate is the standard initial systemic chemotherapy. The addition of rituximab, monoclonal antibody against CD-20 antigen, has also shown benefit and is well tolerated [20]. Nevertheless, mortality rates for PIOL are variable in literature because of inconsistent patient populations and the range is between 9%-81% [9].

## Conclusions

In conclusion, ocular lymphoma and toxoplasmosis are the competing differential diagnosis for patients with retinal whitening and vitreous opacity. Diagnosis is challenging and is often made with delay. Now as ibrutinib is commonly being used for CLL management and there are some concerns of increased risk of opportunistic infections, it is crucial to distinguish these two pathologies early as the management will be completely different.

## References

[1] Butler NJ, Furtado JM, Winthrop KL, Smith JR (2013). Ocular toxoplasmosis II: clinical features, pathology and management. Clin Exp Ophthalmol.

[2] Su RJ, Said J (2019). Intraocular toxoplasmosis mimicking vitreous lymphoma. Blood.

[3] Mastropasqua R, Thaung C, Pavesio C (2015). The role of chorioretinal biopsy in the diagnosis of intraocular lymphoma. Am J Ophthalmol.

[4] Ozgonul C, Besirli CG (2017). Recent developments in the diagnosis and treatment of ocular toxoplasmosis. Ophthalmic Res.

[5] Holland GN (2004). Ocular toxoplasmosis: a global reassessment: part II: disease manifestations and management. Am J Ophthalmol.

[6] Moorthy RS, Smith RE, Rao NA (1993). Progressive ocular toxoplasmosis in patients with acquired immunodeficiency syndrome. Am J Ophthalmol.

[7] Brown JR (2018). How I treat CLL patients with ibrutinib. Blood.

[8] Issa N, Arbona-Haddad E, Nevett-Fernandez A (2017). Opportunistic infections (OIs) in patients with hematologic malignancies (HM) treated with Bruton’s tyrosine kinase (BTK) and phosphoinositide 3 kinase (PI3K) inhibitors: an 8-year retrospective cohort study. Open Forum Infect Dis.

[9] Sagoo MS, Mehta H, Swampillai AJ, Cohen VML, Amin SZ, Plowman PN, Lightman S (2014). Primary intraocular lymphoma. Surv Ophthalmol.

[10] Ongkosuwito JV, Van der Lelij A, Bruinenberg M (1998). Increased presence of Epstein-Barr virus DNA in ocular fluid samples from HIV negative immunocompromised patients with uveitis. Br J Ophthalmol.

[11] Shen DF, Herbort CP, Tuaillon N, Buggage RR, Egwuagu CE, Chan CC (2001). Detection of Toxoplasma gondii DNA in primary intraocular B-cell lymphoma. Mod Pathol.

[12] Chan CC (2003). Molecular pathology of primary intraocular lymphoma. Trans Am Ophthalmol Soc.

[13] Baehring JM, Androudi S, Longtine JJ, Betensky RA, Sklar J, Foster CS, Hochberg FH (2005). Analysis of clonal immunoglobulin heavy chain rearrangements in ocular lymphoma. Cancer.

[14] Hiemcke-Jiwa LS, ten Dam-van Loon N, Leguit RJ (2018). Potential diagnosis of vitreoretinal lymphoma by detection of MYD88 mutation in aqueous humor with ultrasensitive droplet digital polymerase chain reaction. JAMA Ophthalmol.

[15] Batchelor TT (2019). Primary central nervous system lymphoma: a curable disease. Hematol Oncol.

[16] Rajput R, Denniston AK, Murray PI (2018). False negative Toxoplasma serology in an immunocompromised patient with PCR positive ocular toxoplasmosis. Ocul Immunol Inflamm.

[17] Holland GN, Lewis KG (2002). An update on current practices in the management of ocular toxoplasmosis. Am J Ophthalmol.

[18] Jasper S, Vedula SS, John SS, Horo S, Sepah YJ, Nguyen QD (2017). Corticosteroids as adjuvant therapy for ocular toxoplasmosis. Cochrane Database Syst Rev.

[19] Margolis L, Fraser R, Lichter A, Char DH (1980). The role of radiation therapy in the management of ocular reticulum cell sarcoma. Cancer.

[20] Chamberlain MC (2015). High-dose methotrexate with or without rituximab in newly diagnosed primary CNS lymphoma. Neurology.

